# Being active after hip fracture; older people’s lived experiences of facilitators and barriers

**DOI:** 10.1080/17482631.2018.1554024

**Published:** 2019-01-09

**Authors:** Birgit Rasmussen, Claus Vinther Nielsen, Lisbeth Uhrenfeldt

**Affiliations:** a Department of Rehabilitation, Horsens Regional Hospital, Horsens, Denmark; b Department of Public Health, Aarhus University, Aarhus, Denmark; c Faculty of Nursing and Health Science, Nord University, Bodø, Norway; d Department of Health, Science and Technology, Aalborg University, Aalborg, Denmark

**Keywords:** Barriers, facilitators, hip fracture, physical activity, meaningfulness, well-being, older people, qualitative, community, rehabilitation

## Abstract

Hip fracture (HF) incidents can severely restrict the activity and well-being of older people. While participation in activities may be related to lived experiences of meaningfulness, the aim of this study was to explore facilitators and barriers for being active as experienced by older people during the first six months after HF. The study used a phenomenological-hermeneutic methodology informed by the philosophies of Heidegger and Gadamer. Two men and 11 women with reduced functioning prior to the HF were interviewed 2 weeks (*n*=13) and again 6 months (*n*=11) after discharge. Referring to own pre-understanding including a theoretical framework of well-being, a method of meaning condensation was applied to structure the data. A deeper understanding was gradually achieved through a movement between the parts and the wholes. Two themes emerged: (1) “Inner dialogue and actions” with the sub-themes “Inner driving forces” and “Inner limitations”; (2) “Struggling and Striving” with the sub-themes “Building relationships” and “Considering complications and conditions”. We conclude that facilitators for older people to experience well-being while being active involve meaningful relationships with other people, a sense of own identity and being at peace and may be influenced by relationships with staff, physical surroundings, public health services, and health problems.

## Introduction

### Older people´s lived experiences

Older people’s lived experiences hold valuable knowledge about facilitators and barriers for being an active part of their being-in-the-world (Heidegger, 1962) after a hip fracture (HF). Their experiences are holistic and complex, expressing the meaningfulness of being active in the practical everyday life full of interrelationships (Dahlberg, Todres, & Galvin, 2009), and the interdependence of older people and the surrounding world (Heidegger, 1962) implies that after an HF, they are active in a world that is familiar in its everydayness while caring about their future, other people and their physical surroundings. They not only rationally divide experiences into physical, emotional, and social categories, but think freely in a unified experience of meaning. Focusing on the life-world, a good life is possible also during illness. This study uses a theoretical framework developed by Todres and Galvin. Inspired by Heidegger’s writings they describe health as an existential experience of well-being, which at its deepest core is a felt unity of dwelling and mobility (Todres & Galvin, 2010). Dwelling is feeling grounded in the present moment allowing for whatever is there to be; whereas mobility is a kind of restless calling of possibilities, carrying a sense of energy and vitality (K. T. Galvin & Todres, 2011). However, a basic condition of human life is that experiences of well-being exist intertwined with experiences of suffering. Existential suffering can be an experience of being exiled, imprisoned, and unable (Todres & Galvin, 2010), a feeling of anxiety and homelessness in one’s own life, which may be present when illness occurs. This can be a call for action and bring mobility to life as a positive experience, felt as openness towards changes in life while aiming for homecoming to a sense of one’s own identity and an experience of belonging.

### Hip fractures

HF is a sudden traumatic experience from which 40–60% of older people with pre-fracture mobility restrictions never recover (Gustafsson, Gustafsson, Nyman, Bergentz, & Norlin, 2013; Peeters et al., 2016). They seemingly live an inactive life in hospital, during rehabilitation, and still at 6 months after discharge (Davenport et al., 2014; Fleig et al., 2016; Peiris, Taylor, & Shields, 2013; Resnick et al., 2011), which could potentially pose a threat to their well-being and maintenance of physical and mental capacities (Lee et al., 2012; World Health Organization, 2010). A hospital stay likely to be short aims to accelerate older people’s mobility after HF (Giannoulis, Calori, & Giannoudis, 2016) in a fast-track programme (Kristensen & Kehlet, 2012) and by post-discharge community-based rehabilitation in their own home or in a rehabilitation centre (Handoll, Sherrington, & Mak, 2011). Still, 6 months after HF, older people may experience that possibilities for being active are limited when progress is slowing down or ending and loss of functioning still prevails (Ortiz-Alonso et al., 2012).

In Denmark, possibilities for being active after HF areis supported by tax-financed public services including a rehabilitation plan (Kronborg, Bandholm, Kehlet, & Kristensen, 2015) implemented in the municipalities after discharge; access to cost-free loans for aids—for example, a single walker; and modifications in the home such as ramps over doorsteps (Description of rehabilitation efforts, 2010). Further, aiming to maintain older people’s physical capability, social relations and well-being, municipalities in Denmark offer partly or completely tax-financed activities at, for example, day-care centres. However, to our knowledge no studies investigated older people’s experiences of their well-being and possibilities for being active after HF within this context.

### Being active after HF: barriers and facilitators

During hospitalization with HF, older people’s experiences of barriers for being active may be connected with frequency and severity of symptoms such as pain and weakness, and when they need help and staff are missing, they do not ask for help (Brown, Williams, Woodby, Davis, & Allman, 2007). When vulnerability is not acknowledged by the staff, this may add to their suffering; and worries about pain, tiredness, limited mobility, and lack of self-confidence are experienced to be a barrier to a sense of well-being in activity (Perry et al., 2012; Uhrenfeldt & Høybye, 2015)

Loss of functioning and increased dependency, common after HF (Peeters et al., 2016), are in the older population perceived as barriers to being active (Franco et al., 2015). Fear of falling (FOF), prevalent in the lives of older people after HF and for 58% a barrier to be considered after 6 months (Jellesmark, Herling, Egerod, & Beyer, 2012), is a serious condition that can lead to avoidance of activity (Deshpande et al., 2008) and affect mobility (Scheffer, Schuurmans, van Dijk, van der Hooft, & de Rooij, 2008). Being confined to the home by FOF or due to barriers in the physical environment can negatively affect activity and recovery (Bower, Wetherell, Petkus, Rawson, & Lenze, 2016; Moran et al., 2014).

Experiences of well-being seem to facilitate activity which for older people living in the community may be connected with being with peers, feeling safe, and receiving social support, while striving for well-being, better health, and independency (Baert, Gorus, Mets, Geerts, & Bautmans, 2011; Franco et al., 2015). The meaningfulness of being active seems to be vital when aiming for the improvement of well-being, functioning, and participation in social life. A systematic review on self-efficacy and well-being after HF describes well-being to be connected with a sense of being in control, feeling part of a community, and being able to manage in everyday life; whereas receiving help when dependent after HF is experienced as causing worry but also increasing a sense of being able (Rasmussen & Uhrenfeldt, 2016). For older people, a strong sense of identity and being respected as an individual in their relationship with helpers seems to decrease anxiety, helplessness, and feelings of being worthless (Bridges, Flatley, & Meyer, 2010; Gregory, Mackintosh, Kumar, & Grech, 2017; Holm & Severinsson, 2013).

Earlier qualitative studies describe older people’s experiences of participation in exercise and rehabilitation (Resnick et al., 2007, 2005; Tung, Cooke, & Moyle, 2013; Wykes, Pryor, & Jeeawody, 2009), their mobility levels (Taylor, Barelli, & Harding, 2010), and promotion of recovery (Griffiths et al., 2015; Robinson, 1999). However, taking an existential perspective, it is in activities in the familiarity of everyday life that people find meaning in being active (Heidegger, 1962). This study is initiated by four communities and a non-university hospital calling for a physiotherapist (first author) to acquire local knowledge of older people´s experiences of being active after HF. The aim is to explore facilitators for and barriers to being active experienced by older people during the first 6 months after HF surgery.

## Methods

### Design

Choosing a phenomenological-hermeneutic design (Heidegger, 1962), we use individual semi-structured repeated interviews, collecting data at 2 weeks and 6 months after discharge after HF surgery (Brinkman & Kvale, 2015; Kvale & Brinkmann, 2009). The study is the initial part of a longitudinal study interviewing the same participants 4 times, exploring how the meaningfulness of being active may change over the course of 18 months. The design is influenced by Gadamer´s thoughts on the hermeneutic circle as a process of coming to understand through interpretation continuously moving between the parts and the whole of data expanding our previous understanding (Gadamer, Weinsheimer, & Marshall, 2004). Within this methodology, interpretation is taken to be unavoidable, imbedding prejudices in the process. This requires that we as researchers avoid letting biases reflect upon our own pre-understanding in a process of openness towards new possible meanings aiming for our individual horizon to be fused with the otherness of the participants’ experiences into a new understanding.

### Participants

Participants were included (*n *= 9) at the non-university hospital initiating the study. Due to a delay because of few admissions, participants from a similar size and admission-area non-university hospital were included (*n *= 4). Criteria for inclusion were: aged older than 65 years with pre-HF limitation of functional ability, not living in a nursing home, no other actual fractures than HF, and able to speak about experiences in Danish. Gatekeepers were physio- or occupational therapists providing participants with information sheets about the study and obtaining signed informed consent (World Medical Association, 2001). When possible, the first author contacted the participant prior to discharge to initiate a personal contact, offer further information, and settle on a time and place for the interview. Thirteen participants from seven different municipalities were included in the study. Due to 1 participant’s death and 1 participant being in a mentally difficult situation leading to drop out, 11 participants were interviewed at 6 months.

### Data collection

The individual semi-structured interviews were recorded on an Apple® smartphone using the Apple app Memos. At 2 weeks, interviews took place in the participants’ own homes (*n *= 10), at a temporary residence in a rehabilitation centre (*n *= 2), and a day centre (*n *= 1). At 6 months, interviews took place in participants’ own homes (*n *= 10) and in a nursing short-term room (*n *= 1). The first semi-structured interview-guide was developed based on the authors pre-understanding of older people’s situation after HF and further inspired by a theoretical framework of personal well-being (Galvin & Todres, 2011). The second interview-guide took an open life-world approach, allowing the participants to talk more vividly about everyday experiences that were important for them and how they experienced their activities. For example, rather than asking “What makes you feel like being active?” during the first interview round, questions such as “When do you feel comfortable?” were posed in the second interview round. At both interviews, questions were open-ended; probing and reflecting back aimed at letting a deeper meaning emerge, whereas silence allowed participants’ new thoughts to emerge. Some participants were of few words and closed questions were used while paying attention to non-verbal expressions such as body language and tone of voice. To establish a positive atmosphere, the interviewer listened actively by keeping eye contact and nodding to encourage the participant to keep talking (Brinkman & Kvale, 2015). Immediately after each interview, notes about impressions and situations were made.

### Data analysis

The two interview rounds were collected for a mutual analysis. The analysis of the data from the first interview round expanded the researchers’ horizon and sensitized the search for similarities, contrasts, and developments in the second interview round, leading to common themes. A predetermined, structured five-step movement was used to organize and analyse the data (Brinkman & Kvale, 2015), requiring the first author’s immersion in the data for a period of approximately 18 months and progressively focus on the emergence of themes. Interpretation was a circular movement between the five steps, ensuring credibility when a new understanding of the parts—for example, of one interview—was reflected in a better understanding of the whole—that is, all interviews—and vice versa. Firstly, interviews and notes were listened to and reread several times to get an overall sense of the participants’ historical accounts. Secondly, the first author selected meaning units relevant to the research question as expressed by the participants and condensed them into essential statements. Thirdly, a description produced from the underlying meaning of the condensed meaning units rephrased the participants’ own choice of words leading to step four: interpretation in relation to the research question. A deeper understanding beyond what was actually said was achieved. Rather than coding, keywords pointing to an existential meaning were attached, pointing towards step five: development of themes and subthemes (Brinkman & Kvale, 2015). Establishing dependability and confirmability through peer validation, reflections with co-authors on preliminary themes were leading to revising and refining the interpretation (Fleming, Gaidys, & Robb, 2003). We chose citations to confirm themes representing older people’s experiences during the first 6 months after HF. The researchers contrasted the theoretical framework of well-being with the preliminary description of themes to reach a further understanding at an existential level.

### Ethics

The study was registered in the Central Denmark Regional Research Council journal no. 1–16-02–422-15. Gatekeepers introducing the study and study-handout shielded participants from the researcher’s eagerness to include them. Prior to each interview, the researcher ensured confidentiality, the option to withdraw from the study at any time, and the possibility to decline to answer any question during the interview. Debriefing after each interview was an opportunity to discover if participants had bad experiences, taking the time to talk in a more informal way and achieving a sense that the participants’ dignity was in place. The above-mentioned steps were taken to avoid causing harm, an issue in old age calling for specific attention (Sarvimaki & Stenbock-Hult, 2016).

## Results

Table I provides an overview of participants. They had a mean age of 74.5 years. Two men and one woman were married; 10 women were widowed for at least 5 years. Participants living alone all had family, neighbours, or a close friend helping out with various practical tasks. After discharge, all participants needed more help than before the HF. At the time of the first interview, eight participants received help from homecare while three participants were temporarily staying in a rehabilitations centre. At the second interview, 10 out of 11 participants had increased dependency on help or aids than before the HF.

**Table I.** Characteristics of participants.

Code name Age/gender	Interview context (1st/2nd interview)	Walking aid prior to HF	Dependency in daily activities prior to HF	Illness mentioned by participants	Housing conditions/family status
Dorthe 73/F	Own home/own home	Walker outdoor	Cleaning. Medicine dosage	Difficulties speaking from stroke 10 years ago; diabetes; fracture of left upper arm	Old-age housing, town area/widow, children nearby
Hannah 77/F	Temporary rehab stay/own home	Walker outdoor	None	Almost blind with decreasing eye sight due to calcification of the retina; injured nerve in leg; osteoarthritis in the back	Villa, town area/widow, children nearby
Ingrid 77/F	Own home/dropout due to death	Walker	Cleaning	Heart attack; recently completed rehabilitation after spinal cord injury surgery resulting in paralysis of legs; use catheter	Villa, town area/married, unclear where children live
Bodil 78/F	Own home/own home	Walker	Shower. Cleaning. Medicine dosage	Recently completed rehabilitation after stroke with reduced function of right leg; anaemia; dizziness; kidney problems	House, countryside/widow, living with son and daughter-in-law
Karen 80/F	Own home/own home	Walker	Shopping	Parkinsonism; reduced function of first and second finger after surgery of the shoulder; contra lateral HF 5 years ago; dizziness	Ground level flat, town area/widow, children nearby
Margrethe 80/F	Own home/own home	Walker outdoor	None	Chronic headache; dizziness; cancer surgery	Apartment, 6 steps up, town area/widow, children far away, male friend nearby
Anna 85/F	Own home/nursing short term room	Walker	Compression stockings	Gout of fingers and neck; pneumonia at time of HF	Old-age housing, town area/widow, children nearby
Joan 85/F	Own home/own home	Walker away from own plot	Security visits. Cleaning	HF	Villa, town area/widow, children nearby
Nelly 89/F	Own home/dropout due to mental state	Support from furniture or person	Medicine dosage. Cooking. Cleaning. Shopping	Almost blind with decreasing eyesight due to diabetes	Apartment, 6 steps up, town area/widow, children nearby
Lene 90/F	Own home/own home	Walker outdoor	Cleaning	Chronic obstructive lung disease	Old-age housing, town area/widow, children far away
Else 92/F	Temporary rehab stay/own home	Walker	Cleaning. Laundry. Medicine dosage	Almost blind with decreasing eyesight due to diabetes	Villa, town area/widow, children far away
Frank 70/M	Day-care centre where he stays every weekday/own home	Wheel chair, walker, four-point cane	Transfer. Personal hygiene. Dressing. Wife manages the house	Completed stroke rehabilitation 9 months ago. Right-sided drop foot. Not able to use right hand. Cancer successfully treated. Repeated surgical removal of fistulas in the brain	Villa town area/married, son living at home
Gunnar 77/M	Temporary rehab stay/own home	Walker, support from wife	Transfer. Wife manages the house	Reduced balance and memory from stroke; several herniated discs; spinal stenosis	Villa, town area/married, children nearby

Experiences during hospitalization seemed to be in the background and could be hard to remember; descriptions tended to be undetailed, except experiences of suffering that made an impression.

To explore facilitators for and barriers to being active among older people during the first 6 months after being discharged after HF surgery, we identified two main themes in the data analysis: “Inner dialogue” and “Struggling and striving”.

### Inner dialogue

Unable to manage independently after HF and resume their previous level of activity, participants felt “homeless” and unsafe in activity. To feel and return to a sense of well-being and familiarity with their life-world, they had an ongoing inner dialogue between their “Inner driving forces” and “Inner limitations”. They were moving between openness towards possibilities and lost hope of overcoming limitations.

#### Inner driving forces

A sense of belonging was a driving force related to sharing and being in this together with other people, having a feeling of being connected with and still belonging to the world. When the body was not able to carry out meaningful everyday life projects, participants were reaching for a normalization of their life. Sharing practical tasks and problems, newspapers, and celebrations brought joy, contentment, and the value of progress. Through long relationships, family members knew their preferences and routines, and Lene explained how they took care of “small things you normally do not count, but just do. But in my present situation they are big things.” Particularly for participants recently discharged and isolated in the home, visits were energizing; and for Joan, friends visiting made her feel that “they haven’t forgotten you”. Experience of change and something new to life gave participants the energy to continue. Going to social events or a day-care centre was a welcome break from the everyday boring routine, and participants even felt they belonged to a community. Bodil was feeling lonely in her home but “the two days I´m at the day-care centre, they are doing it for me, it’s like an oasis to get there”. To have a sense of belonging was a confirmation of being alive and gave feelings of hope and meaningfulness.

A sense of identity was a driving force related to being able to do things independently and feeling dignified. Participants were aiming for homecoming in a sense of being in touch with personal capacities and values. Being persistent, creative, positive, vigilant, and thoughtful supported a sense of being, and experiences of progress maintained hope and self-confidence. Responsibility was a matter of finding solutions to problems and having duties. Karen had managed baking cookies for my second visit:

I tell myself, you HAVE to try, and then when a full baking sheet is ready, I go and sit down for a while. Then back to the oven again. It can take a long time but I have nothing but time.

Though at times participants pushed the limits of their capacities, they moved about with care to avoid falling, while balancing a need for being active with a need for rest. Independency and not having to wait for help was even more important than avoiding pain, and participants had a drive towards deciding for themselves. Self-imposed activities of doing household tasks, exercising, going for walks, or participating in social activities brought a sense of dignity. For Joan, this lead to unintentionally offending a woman passing by, who offered to help pick up a pile of gathered up leaves in the driveway:

she was quite offended. I’ve […] told her that it wasn’t that I didn’t want her help, but it was because I needed to do it on my own […]. You tell yourself “You can do it when you have to” and then you do it.

In contrast, being at peace with the situation was a driving force. Participants accepted suffering and limitations as part of life and becoming old; they came to terms with prolonged rehabilitation and dependency on help or assistive devices; abstained from making unrealistic plans; and found meaning and joy in being in their own homes or in good memories. Living with chronic limitations from her Parkinsonism, Karen philosophized that “when you are ill, all you want is to be well again, and when you are well what then; […] then you don’t think about it as much”, indicating how suffering was balanced by appreciating the presence of good experiences.

#### Inner limitations

All participants had experiences of feeling helpless related to a sense of their own identity under pressure. Being unable to do what they longed for was distressing and they were suffering defeats when they wanted to do more than they could handle. The will alone was not sufficient when the body was vulnerable and they were despairing about a loss of their capacities. Margrethe recently stopped attending physical therapy training 6 months after the HF, but recognized then how her pain came back:

“I wanted the pain to stop […] because going to the city, that’s always nice. But I don’t feel like going at all anymore because I can’t walk, […] and I don’t really enjoy a trip like that”.

Primarily at the first interview, participants felt helpless in experiences of undignified dependency, feeling inadequate in not able to get out of bed, or to be a hostess for guests. Their efforts did not necessarily pay off, and at 6 months, slow progress or lack of progress put their hopes under pressure. For Lene, her dream of being active and socializing without worries in old age had shattered:

“ALL the things that I had thought I would be able to do nice and slow […] just isn’t happening. Now, it has become exhausting instead just to get a spoon or […] get something from a shelf”.

Participants felt out of control and in doubt about what to expect. At the first interview, fears were about moving with pain and about how much the leg could withstand. Prior to discharge, the future was blurry and they worried about keeping up steam after discharge and about permanent loss of their ability to walk. Feeling unprotected after discharge, participants had an inner dialogue about avoiding being in a precarious situation to avoid losing control. Staying at home from valued social activities was preferred when it was strenuous to get ready in time, risky to go home in the dark, or depending on other people which meant they could not decide the duration of a visit.

Participants had concerns about becoming a burden. Striving to be in equal relationships, they loathed the idea of laying a burden on family or friends. For Joan, this meant resigning from going to bingo, which used to be her great passion:

you have to get a […] seat, go get the cards and […] you have to go to the bathroom once in a while. […] Then, going a little downhill back to the car again is not so nice. […] you don’t want to inconvenience everyone just because you broke a leg.

However, inner driving forces and inner limitations were intertwined. Participants had a dialogue about how to keep on being active when their hopes were under pressure. For Lene, ordinary tasks had become strenuous because it took her so long to walk from one place to another; for example, bringing a cup of tea to the living room while balancing it on top of the walker; and if she forgot a teaspoon, it was the whole trip again. Rather than despairing, Lene used her creativity:

I made kind of an emergency drawer; if I forget anything. Actually, it is a sewing table […] on wheels. […] I have a knife, teaspoons and […] the essentials. You have to figure something out. You sit there thinking about what you can do about this and that.

Also, Lene at 6 months was distressed about being unable to participate in social activities as before the HF. When I asked whether she could still have a good life she said:

Yes, because then I’ll just make changes to some of the things I CAN do. For example […] these entertainments nights; I can’t dance and jump around, but I can BE there […] see the joy of life other people have. And what they are able to do, even if I’m not able to the same, it’s kind of comforting.

A feeling of helplessness and not being in control was balanced by homecoming to a sense of identity, a sense of belonging, acceptance of limitations and new meaning in joining social activities. When inner driving forces dominated, possibilities for well-being in activity were developed; when inner limitations dominated, suffering and inactivity increased.

### Struggling and striving

Suffering brought on by the HF changed participants’ relation with the surrounding world and themselves. Aiming for homecoming, they were struggling to be active while striving for progress described in two sub-themes: “Building relationships” and “Considering conditions and complications”. Cooperation with staff, public services, and physical surroundings could facilitate or be a barrier for homecoming, whereas complications from health issues were always barriers.

#### Building relationships

Staff facilitated participants’ well-being in activity when the relationships were trusting and cooperative, which meant feeling acknowledged for their efforts while receiving feedback and encouragement. Participants acknowledged staff expertise and followed their advice and recommendations when they felt safe about their training and accepted complications to be normal. Training was meaningful when it was challenging, not too difficult, and addressed and helped to overcome complications. Hanna appreciated how cooperation with physiotherapists built confidence:

I get compliments all the time—that feels nice because I do the right things. […] I get the sense of how it feels to have a leg that doesn’t quite cooperate, how it’s supposed to feel so I can carry on training myself.

In hospital and immediately following discharge, help was experienced to be a prerequisite for being active, mitigating the limiting repercussions from the hip HF. When having someone around, participants dared to do things on their own, like Bodil who, terribly afraid to fall, said that “the reason why I continue to have home care is that I simply don´t dare to be here alone and take a shower”. Taking pride in managing independently, participants struggled to receive only the necessary help and to gradually reduce help in daily life activities.

In contrast, when there was no cooperation with staff but rather orders, suspicion, or neglect, participants felt “homeless” and left on their own. Pain was inflicted when hospital staff demanded swiftness in activities and a fall was the consequence of staff neglecting other diseases than the HF. Anna was dependent on help in her home in the wait for Re-surgery of the hip and felt overlooked when busy staff did not recognize her needs:

and then all of a sudden they forget a lot of things for example when I used a walker, they could forget to put it where I could reach it and there are a lot of things like that. That’s no good.

Participants felt stuck and helpless when the help they needed was not present.

#### Considering complications and conditions

Complications were body and health issues (Table II). They added to the sufferings of participants and deprived them of the joy of being active. At the first interview, though pain was unbearable and required slowness during mobility, it was accepted as natural. Participants loathed drug side-effects and struggled to find a balance between pain and painkillers. Complications deprived participants of their energy and could turn into a vicious circle of growing bodily discomfort requiring inactivity while striving for mobility and a social life. Though complications threatened the well-being of being able to move freely and participate in meaningful activities, apart from Gunnar whose wife continuously took action to secure ongoing supervised training, participants did not ask for additional rehabilitation.

**Table II.** Frequency and type of complication.

Type of complication	Iw. 1^1^	Iw. 2
Lack of physical capacity ^2^	*n *= 10	*n *= 9
Pain	*n *= 10	*n *= 3
Side-effects from medicine	*n *= 8	*n *= 2
Other diseases or disorders	*n *= 7	*n *= 10
The whole leg affected (not only the hip)	*n *= 6	
Fatigue	*n *= 4	*n *= 3
Swelling of leg	*n *= 3	*n *= 2
Movement restrictions	*n *= 3	
Permanent catheter with bag	*n *= 1	
Disturbed sleep	*n *= 1	*n *= 1
Subluxation of the hip	*n *= 1	

^1^ Iw. = Interview

^1^ Lack of strength in the leg, risk of falling, not able to walk, not able to climb stairs, reduced hip mobility, not able to stand on the leg, limping

Conditions were public services (Table III) and the physical surroundings. External settings and surroundings enabled a sense of feeling confident, provided possibilities, and expanded reach and horizon, such as modifications of the home, assistive devices, day-care centres, and public transportation. In contrast, participants felt lost or powerless with inadequate assistive devices or incomplete modifications of the home; or when stuck in the home unable to get around using public transportation. Surroundings could give a sense of homelessness when purpose and possibility for being active seemed lost, such as being in a hospital environment with nothing to do. For Nelly, a nearly blind woman who had recently moved due to a fire in her previous apartment, being in her home was a painful experience of discouragement and imprisonment. When asked about what could make her want to move, she said:

Well I run, walk around here; when I get tired of being in the bedroom I’ll go out here [to the living room]; then I’ll go out into the kitchen […]. If I can’t pass the time in one place, I’ll go somewhere else and then there is nothing to do but wait.

**Table III.** Conditions: available and missing public services.

Facilitators: available public services	Barriers: missing public services
● “The hospital [staff] saw to [it] that I received a rehabilitation plan to start rehabilitation at home.” (Joan)	● “[Before discharge] I wanted to go to a rehab centre so I would not be alone and there was someone to guide me when walking. Hospital staff say it is not possible in my community.” (Anna)
● “I have to go to the doctor with my diabetes. I just call a flex-taxi. I can bring my walker. They are good at helping me.” (Anna)	● “Rehabilitation doesn’t start until three weeks after discharge.” (Frank)
● “I was about to go home, but as long as I depend on help with everything, a bed and toilet in the living room is necessary. However, this doesn’t work. So we chose a rehab centre.” (Else)	● “It’s hard not to be able to do anything for a long time. I didn’t start rehabilitation until after a week. It could have started earlier.” (Ingrid)
● “If I had gone home from hospital without previous modifications in the home, I would feel like being in a prison. In rehab, I hope we have time to find out what is wise to get done at home.” (Hannah)	● “For a walk in the garden, what I miss the most is to have a second walker. I need to bring it all the way down the stairs and back on the carpet with dirt. They took it away from me.” (Else)
● “Being picked up and dropped off and picked up again [to go to day-care centre], that suits me amazingly because people I don’t know, it’s horrible! I’m not sure I would go if I had to do it on my own.” (Bodil)	● “I wanted to walk to the supermarket and had to apply to borrow a walker. It took four weeks before I received it.” (Hannah)
● “We do all kinds of activities at the day-care centre; play cards, watch movies, sometimes bingo, sometimes go out walking together. When the weather is nice, we even go down to the harbour and look around.” (Bodil)	● “Due to the hassle in manoeuvering the walker over the doorstep, I mostly stayed in the bedroom. It wasn’t until two weeks after discharge I had ramps put over my doorsteps.” (Karen)
● “The physiotherapist from rehab went home with me to see to that it was safe to send me home.” (Ingrid)	● “The doorways are too narrow or the walker too broad. Moving the walker through the doorways, I hurt my fingers.” (Lene)
	● “I can walk to the bus, but it’s too far to walk around between shops to buy a new pair of shoes for my compression stockings.” (Lene)

Confined to her home, there was no respite from the boredom of having nothing to do in a restricted space. To go out of the home, they needed the physical surroundings to be supportive. For Joan, stairs were an insurmountable barrier preventing her regular visits next door to a good friend confined to a wheelchair:

It’s been a long time since I’ve been inside because I haven’t had the courage to go around and walk up the stairs no, no. Before, I would have gone up through the kitchen to have a chat. Just to talk for half an hour which means a lot.

Participants planned where and when they would go and preferred the safety of walking near other people, on smooth surfaces, in good weather, and for short distances.

## Discussion

This study investigating older people’s lived experiences of facilitators and barriers for being active after HF confirms evidence on the influence of symptoms, functioning, personal, social, and environmental factors (Baert et al., 2011; Franco et al., 2015), and adding an existential perspective points to the unified experience of facilitators and barriers. Contributing to the framework of well-being (Todres & Galvin, 2010) in showing examples from the life-world of older people with an HF, this study expands the understanding on the intertwined experience of well-being and suffering. The HF was a life-changing event and experiences of well-being made it possible to find meaning in being active and were resources while balancing suffering. Suffering in this study appeared as feelings such as a sense of homelessness, hopelessness, and not being in control in the absence of longed-for activities, and participants were struggling and striving to have a sense of belonging, of identity, and of being at peace. Participants were active, struggling within the boundaries of their physical or environmental limitations. Well-being was possible when a sense of being at home was present or when striving for “homecoming” expanded the possibilities of being active. Depending on whether their inner dialogue was dominated by their inner limitations or inner driving forces, well-being or suffering alternatingly were in the foreground of experiences, reinforced by the relationships they were building with staff. When participants felt recognized by staff for their effort and not only the results, their self-confidence increased; feeling recognized may be fundamental when benefitting from staff expertise (Rasmussen & Uhrenfeldt, 2016) and information received after HF (McMillan, Booth, Currie, & Howe, 2012). In contrast, feeling treated as an object when orders and suspicions were part of older people’s relationships with staff could raise a concern about being able and lead to a downslide into suffering. This is possible when a range of small incidents pushes towards a feeling of being unable, and losing courage may be the result (Uhrenfeldt & Høybye, 2015). While treating older people as experts on their own health, respecting their needs, problems, and desires in life (Holm & Severinsson, 2013) may support them in being active and overcoming suffering.

The framework of well-being described a sense of identity as a feeling of being able, an unspoken feeling of optimism (Galvin & Todres, 2011); this study added the understanding that a sense of identity was not only a state older people were in, but a dynamic inner dialogue concerning possibilities and limitations for being active. It was a resource connected with a sense of self-confidence and the possibility for progress while keeping on making plans and carrying out tasks and duties. A sense of identity seems to be closely related to independency and possibilities for making one’s own decisions (Holm & Severinsson, 2013) and even possible when being in a dependent situation (Rasmussen & Uhrenfeldt, 2016). However, feeling unable while being dependent after HF, a sense of identity was under pressure and participants had feelings of despair. Galvin and Todres (2012) point to how a sense of not being able is linked with feeling useless, and how withdrawal into isolation and inactivity may be the consequence. To fully understand whether the individual’s experience of well-being in activity after HF is compromised, attention towards a potential split between what they want to be able to accomplish and feelings of helplessness (Galvin & Todres, 2012) is called for. Helplessness and despair may deprive them of a sense of energy (Galvin & Todres, 2011), whereas involvement and increased feelings of being in control in activities that are wanted may increase older people’s agency (Gregory et al., 2017).

Feeling safe and supported by the physical home, the outdoor environment (Moran et al., 2014), and availability of public services, participants were active in a sense of comfort and well-being. After HF, the home, the streets, and the journey from home to other places may become places of inhospitability, feeling "homeless" and insecure when moving (Todres & Galvin, 2010), and anything that can modify the inhospitable constraint in the environment and improve a sense of freedom to move without a risk of falling may facilitate well-being and activity. Further, being confined to the home in the early phase after discharge, participants in this study felt isolated with nothing to do; an experience of immense suffering and inactivity.

Evidently older people in this study did not recover from the HF and they were struggling to be active within the limitations from complications and conditions. Some of their health problems may be modifiable; FOF seems possible to reduce through prolonged rehabilitation (Dukyoo, Juhee, & Lee, 2009; Ziden, Frandin, & Kreuter, 2008) and it is argued that progressive rehabilitation programmes targeting deficits in balance, strength, and mobility after HF give older people the best chance of avoiding impaired functioning (Sims-Gould, Stott-Eveneshen, Fleig, McAllister, & Ashe, 2017). However, participants did not seek assistance. Not seeking help may be connected with a sense of meaning in activity being maintained from being able to manage independently and deciding for themselves. Other studies point to a lack of knowledge about services (Gregory et al., 2017) or preserving a sense of dignity. There seems to be a risk of overlooking possibilities for further improving older people’s mobility and well-being in activity after HF.

### Implication for practice

Approaching older people’s own needs and wishes, emphasizing how homecoming to a sense of belonging, to a sense of one’s own identity, as well as to a realization that acceptance is possible, may increase their possibilities for being active. Additionally, we suggest that hospital staff members consider whether a feeling of belonging is possible after discharge; without meaningful relationships that are practical and vitalizing, and a confirmation of being precious to other people, a discharge directly to the participant’s home may not be the best solution. Further, community guidelines and community staff may secure a called-for continuous proactive attention towards older people’s hidden needs for further interventions beyond the end of rehabilitation.

### Methodological consideration

It could be a limitation of this study that only 13 participants were included and only 11 participants still remained in the study at the second interview. However; analysing the large amount of data from 26 hours of interviews is time-consuming and adding more participants may have led to superficial analysis without capturing the depth of individual experiences (Brinkman & Kvale, 2015). Rather than demographic variation, depth is the aim within phenomenological-hermeneutic research (Norlyk & Harder, 2010). Still, participants represent variation in age, sex, dependency prior to and after the HF, family relationships, and residential status, adding details and richness to the analysis.

The pre-understanding of the first author being a physiotherapist caring about improving functioning and independency after HF may have influenced the findings. However, congruent with the phenomenological-hermeneutic methodology through discussions, the interdisciplinary research team identified biases from prejudices and developed a deeper understanding (Fleming et al., 2003).

The study was undertaken within the tax-financed Danish healthcare system. Depending on recognizability of the individual situation and context (Shenton, 2004), results from the study may be transferable to older people suffering from recent loss of functioning due to other diseases; for example, after long-term, geriatric hospital stays. The two men in the study both had wives helping out and taking care of everyday tasks, while men living by themselves may have different experiences of being active.

In three cases, the presence of relatives at the interviews might have been a limitation but was ultimately experienced to be a strength. Regarding ethical concerns, they were not asked to leave as participants’ situations are considered part of a reciprocal relationship with close relatives (Haahr, Norlyk, & Hall, 2014); instead, the researcher underlined that the focus of the interview was the participants’ experiences. On two occasions, the presence of a son and a wife seemed to provide a sense of comfort for the participants. Further, the relatives’ presence was judged to present a foundation on which the individual experiences of the participants stood out more clearly (Gadamer, 2004). Participants who had previously suffered a stroke occasionally had difficulties elaborating on topics; specific attention towards their comfort during the interview involved repeatedly selecting questions about action considered less intrusive than questions about beliefs and values (Price, 2002). Further, the interviewer occasionally suggested directions for answering questions about meaning. This was a limitation and the researcher took special precautions during analysis to include only suggestions that seemed meaningful for participants: the non-verbal communication – for example, their tone of voice – and the knowledge about participants from repeated interviews was considered.

## Conclusion

This study provides knowledge regarding the existential nature of older people’s experiences of facilitators for and barriers to being active after HF, increasing the understanding of how well-being may be possible and suffering diminished during the first 6 months. Two themes describe how a sense of coming and being at “home” is a facilitator and possible when suffering is balanced in an inner dialogue between personal driving forces and limitations. After HF, older people are struggling and striving, and relationships with staff, conditions, and complications influence their well-being. Non-demanded help from health care professionals may be relevant to avoid older people sliding into feelings of helplessness and being unable after HF. Possibilities for well-being may be facilitated through enhanced inner driving forces, environment- and health-improvement also beyond 6 months after HF. Future research taking an existential approach to gain more detailed knowledge on how well-being is possible as a resource for older people after HF during hospitalization and during specified interventions in communities is needed.
